# Ligand Selectivity in the Recognition of Protoberberine Alkaloids by Hybrid-2 Human Telomeric G-Quadruplex: Binding Free Energy Calculation, Fluorescence Binding, and NMR Experiments

**DOI:** 10.3390/molecules24081574

**Published:** 2019-04-21

**Authors:** Nanjie Deng, Junchao Xia, Lauren Wickstrom, Clement Lin, Kaibo Wang, Peng He, Yunting Yin, Danzhou Yang

**Affiliations:** 1 Department of Chemistry and Physical Sciences, Pace University, New York, NY 10038, USA; yy86950n@pace.edu; 2 Department of Mathematics and Department of Research Computing, Princeton University, Princeton, NJ 08544, USA; junchao.xia@princeton.edu; 3 Department of Science, Borough of Manhattan Community College, the City University of New York, New York, NY 10007, USA; lauren.wickstrom@gmail.com; 4 Department of Medicinal Chemistry and Molecular Pharmacology, College of Pharmacy, Purdue University, West Lafayette, IN 47907, USA; lin835@purdue.edu (C.L.); wang3318@purdue.edu (K.W.); 5 James Frank Institute, and Institute for Biophysical Dynamics, The University of Chicago, Chicago, IL 60637, USA; hepeng.yanglu@gmail.com

**Keywords:** G-quadruplex, protoberberine, NMR, molecular dynamics simulation, binding free energy, MM-PB(GB)SA, NMR titration

## Abstract

The human telomeric G-quadruplex (G4) is an attractive target for developing anticancer drugs. Natural products protoberberine alkaloids are known to bind human telomeric G4 and inhibit telomerase. Among several structurally similar protoberberine alkaloids, epiberberine (EPI) shows the greatest specificity in recognizing the human telomeric G4 over duplex DNA and other G4s. Recently, NMR study revealed that EPI recognizes specifically the hybrid-2 form human telomeric G4 by inducing large rearrangements in the 5′-flanking segment and loop regions to form a highly extensive four-layered binding pocket. Using the NMR structure of the EPI-human telomeric G4 complex, here we perform molecular dynamics free energy calculations to elucidate the ligand selectivity in the recognition of protoberberines by the human telomeric G4. The MM-PB(GB)SA (molecular mechanics-Poisson Boltzmann/Generalized Born) Surface Area) binding free energies calculated using the Amber force fields bsc0 and OL15 correlate well with the NMR titration and binding affinity measurements, with both calculations correctly identifying the EPI as the strongest binder to the hybrid-2 telomeric G4 wtTel26. The results demonstrated that accounting for the conformational flexibility of the DNA-ligand complexes is crucially important for explaining the ligand selectivity of the human telomeric G4. While the MD-simulated (molecular dynamics) structures of the G-quadruplex-alkaloid complexes help rationalize why the EPI-G4 interactions are optimal compared with the other protoberberines, structural deviations from the NMR structure near the binding site are observed in the MD simulations. We have also performed binding free energy calculation using the more rigorous double decoupling method (DDM); however, the results correlate less well with the experimental trend, likely due to the difficulty of adequately sampling the very large conformational reorganization in the G4 induced by the protoberberine binding.

## 1. Introduction

**Figure 1:**
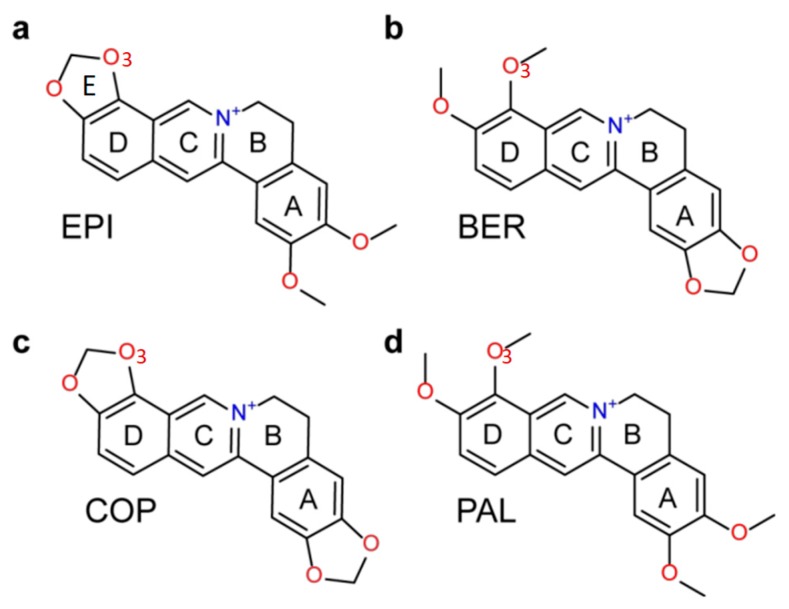
Chemical structures of protoberberine alkaloids: (**a**) epiberberine (EPI), (**b**) berberine (BER), (**c**) coptisine (COP) and (**d**) palmatine (PAL).

G-quadruplex DNA is a four-stranded non-canonical secondary structure formed in DNA sequences containing consecutive runs of guanines such as d(TTAGGG)_n_ in human telomeric DNA. A G-quadruplex consists of stacked planar building blocks called G-tetrads, containing four guanines connected by a network of Hoogsteen hydrogen bonding [1]. To form stable G-quadruplex, monovalent cations K^+^ (or Na^+^) centrally located between the adjacent G-tetrad planes are required [2]. The human telomere is a region of repetitive nucleotide sequences at the ends of chromosomes, which protects the chromosome from degradation [3]. In normal cells, each cell replication results in a shortening of the telomere, which will lead to apoptosis or programmed cell death when a critical shortening is reached. However, in cancer cells the length of the telomere is extended by telomerase, which maintains the malignant phenotype by stabilizing telomere length [4]. Intramolecular G-quadruplex formed by the guanine-rich DNA sequences d(TTAGGG)_n_ in telomeres inhibits the telomerase access, and G-quadruplex-interactive small molecules have been shown to stabilize the G-quadruplex structures and inhibit telomerase activity, as well as disrupt telomere maintenance [5,6]. The natural product protoberberines are a class of alkaloids that interact with nucleic acids and have anticancer activities [7]. Studies have shown that berberine and its derivatives inhibit telomerase and have selectivity for the human telomeric G-quadruplex relative to duplex DNA [8]. We have previously found that among the several structurally similar protoberberines, epiberberine (EPI) (Figure 1a) exhibits the greatest fluorescence enhancement upon binding to human telomeric G4 in K^+^ solution, up to 45 times stronger than other G4s and double-stranded DNA [9]. However, it was unclear how the alkaloids bind the human telomeric G4, as the structures of both hybrid-1 and hybrid-2 human telomeric G4s contain no obvious binding pocket to accommodate the large tetracyclic alkaloids, with both the 5′- and 3′-ends of the DNA occupied by disordered flanking segments. This has been solved by our recently published NMR study, which reveals that EPI recognizes specifically the hybrid-2 form of the human telomeric G4, and most strikingly, upon binding, EPI converts other forms of the telomeric G4s to the hybrid-2 structure [6]. The NMR structure of the EPI in complex with the hybrid-2 form of the human telomeric G4 shows that the EPI binding induces complete rearrangements of the 5′-flanking segment and loop regions leading to the formation of a highly extensive four-layered binding pocket at the 5′ end of G4 [6]. Interestingly, the three structurally similar protoberberines, berberine (BER), palmatine (PAL) and coptisine (COP) (Figure 1), which share the same heterocyclic core and differ only in the positions of a methylenedioxy moiety and two methoxy groups located at the two ends, bind the hybrid-2 telomeric G4 much weaker than EPI [9]. While the NMR structure provides some clues for superior binding of EPI, it only provides a qualitative explanation but not the quantitative measure of the binding affinity. Moreover, it is a static structure whereas at room temperature molecules undergo constant thermal motion in solution. It is therefore important to examine the ligand selectivity when the thermal fluctuation in conformational dynamics is taken into account.

To more quantitatively examine the molecular recognition of protoberberines by the human telomeric G4, in the present study we employ two molecular dynamics binding free energy methods, MM-PB(GB)SA [10,11,12] (molecular mechanics Poisson Boltzmann/Generalized Born surface area) and DDM [13,14,15,16] (double decoupling method) to compute the binding free energies of the four protoberberines for the hybrid-2 human telomeric G4 wtTel26 [2]. We compare the calculated binding free energies with the experimental binding affinities determined in this study using fluorescence. From the energetic decomposition of the computed binding free energies and the MD (molecular dynamics) simulated structures, we seek to gain insights into the mechanism of the ligand selectivity. Ideally, the FEP [17,18] (free energy perturbation) method is best suited to compute the relative binding free energies for structurally similar ligands. However, for the four protoberberines shown in Figure 1a, the chemical modifications that convert one ligand to another require ring breaking. Such perturbations involving chemical bond breaking and creation is known to be difficult to converge in FEP calculations [19], although new method development is beginning to make such calculations more feasible [20]. One challenge in computing the binding free energy for DNA-ligand arises from the fact that the current force fields for simulating nucleic acids are less accurate compared with those used for simulating proteins [21,22]. Here we compute binding free energies using two widely used Amber force fields: parm99bsc0 [23] (bsc0) and the more recent variant parm99bsc0_OL15_ parameters set (OL15) [24]. One of the goals of the present study is to evaluate how well the computational methods employing the current DNA force fields capture the experimental trend of the ligand selectivity and reproduce the NMR structure of the DNA-ligand complex.

## 2. Results and Discussions

### 2.1. The MM-PB(GB)SA Binding Free Energies Calculated from Molecular Dynamics Simulations Correlate Well with Experimental Binding Affinities

**Figure 2:**
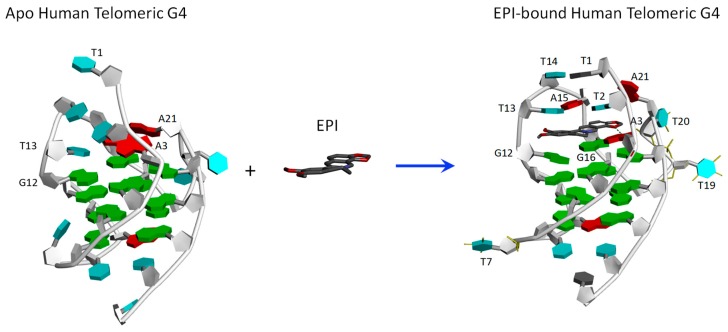
Binding of EPI induces a complete rearrangement of the 5’-end structure of the human telomeric G4 [6].

**Figure 3:**
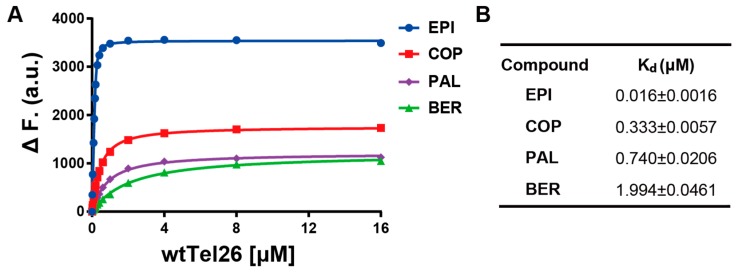
(**A**) Fluorescence intensity of four protoberberines (0.2 μM) upon titration with wtTel26 DNA, showing 1:1 binding by the curve fitting (solid line). Conditions: 25 °C, pH 7, 100 mM K+. (**B**) The determined disassociation constant (Kd) values of the EPI, COP, PAL, BER.

We utilize the NMR structure of the complex of EPI with the wild-type human telomeric G4 sequence wtTel26 (TTA[Q]TT, [Q] = 5′-G_3_(TTAG_3_)_3_-3′) to investigate the molecular mechanism of the ligand selectivity in the recognition of protoberberines by the human telomeric G4. The NMR structure shows that the EPI binding induces complete rearrangements of the 5′ end flanking and loop segments leading to the formation of a highly extensive four-layered binding pocket at the 5′ end of hybrid-2 human telomeric G4 [6] (Figure 2). The four structurally similar protoberberine alkaloids: EPI, berberine (BER), palmatine (PAL) and coptisine (COP), share the same heterocyclic core and differ only in the positions of a methylenedioxy moiety and two methoxy groups located at the two ends (Figure 1). However, the fluorescence binding affinity measurements performed here show that EPI binds wtTel26 at least 20 times greater than the other three protoberberines (Figure 3). The NMR structure provides some clues for superior binding of EPI. For example, in the NMR structure, the EPI forms H-bond (Figure 2) with A3 through its methylenedioxy ring E (Figure 1a); in BER and PAL, the methylenedioxy is replaced by methoxy groups which could weaken the H-bond with A3. In addition, in BER and COP the methylenedioxy ring at the other end may cause steric clashes with the DNA backbone (Figure 2).

We first use molecular dynamics binding free energy method MM-PB(GB)SA to calculate the absolute binding free energy for each ligand. One potential challenge in estimating the binding free energy of protoberberines with the human telomeric G4 could come from the very large conformational change induced by the ligand binding (Figure 2). Here, the more approximate MM-PB(GB)SA has one advantage in that it is an end-point method, which does not need to sample the intermediate states connecting the two end states.

**Figure 4:**
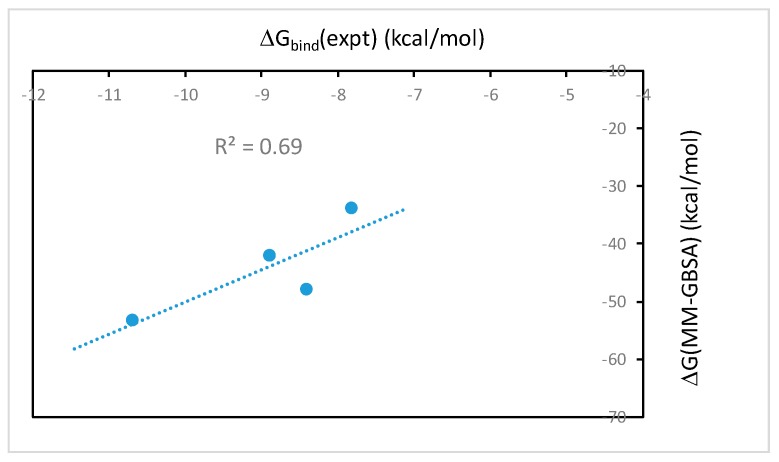
The correlation of the ΔG(MM-GBSA) binding free energies calculated using the bsc0 force field vs. experimental binding free energies of the investigated protoberberines with wtTel26 in K^+^ solution.

The binding free energies of wtTel26 G4 in complex with the four alkaloids using the MM-GBSA method with the bsc0 force field are shown in Table 1. The results of ΔG(MM-PBSA) (data not shown) are essentially the same as ΔG(MM-GBSA). As seen from Table 1, the calculated ΔG(MM-GBSA) for EPI (−53.3 kcal/mol) is the most favorable among the four alkaloids, in agreement with the experimental binding constants (Figure 3). Table 1 also shows that the calculated ΔG(MM-GBSA) for BER is much weaker than the other ligands, again in agreement with the experimental measurements (Figure 3). Figure 4 shows the correlation between the calculated ΔG(MM-GBSA) with the experimental binding affinities. The R^2^-value of 0.69 shows that the computational results correlate well with the experimental data.

### 2.2. The MM-PB(GB)SA Binding Free Energies Calculated Using Single Energy-Minimized Structures Show No Correlation with the Experimental Data

We have also calculated the MM-PB(GB)SA binding free energies using single energy-minimized structure of the ligand-DNA complexes. Such calculations ignore the dynamic effect in receptor-ligand binding, but because it is computationally fast, the protocol is widely used for rapid in silico screening of ligand binding [25]. However, our result shows that there is no correlation between the MM-PB(GB)SA free energy calculated using the single minimized structure and the experimental binding affinities of protoberberine-wtTel26 G4 binding (data not shown). This result is in contrast with the MM-PB(GB)SA binding free energies calculated using snapshots from molecular dynamics simulations, which show good correlation with the experiments (Figure 4). These results therefore demonstrate that the dynamic ligand-DNA conformational coupling plays a crucial role in the ligand selectivity of the protoberberine recognition by the wtTel26 G4. While the single minimized structure MM-PB(GB)SA protocol may work for ligand binding with more rigid binding sites found in many protein-ligand systems, the ligand-DNA complexes in this report are quite flexible such that molecular dynamics sampling is clearly needed to account for the conformational flexibility in order to capture the experimental trend in the ligand selectivity.

### 2.3. MM-PB(GB)SA Free Energy Calculated Using MD with OL15 Force Field also Reproduced Experimental Trend Well but the Simulations Show Structural Distortion in the Protoberberine Binding Site

To examine how the calculated binding free energies depend on the choice of the DNA force field, we performed the molecular dynamics MM-PB(GB)SA calculations using the more recent DNA force field OL15 [24], which contains more refined sugar-phosphate backbone torsion and the χ glycosidic torsion parameters that improved the simulation of the Z-DNA and B-DNA. As shown in Table 2, the ΔG(MM-GBSA) binding free energies calculated using OL15 also correctly ranked EPI as the top binder, and the ranking order of the ΔG(MM-GBSA) is the same as that obtained using bsc0 (Table 1). However, the differences in the ΔG(MM-GBSA) binding free energies separating strongest binders (EPI) and other compounds are substantially smaller than those obtained with the bsc0 force field parameters set: See Table 1 and Table 2. For example, the ΔG(MM-GBSA) for EPI and PAL calculated using bsc0 differ by −5.4 kcal/mol, whereas the same quantities calculated using OL15 differ by just −2.4 kcal/mol. 

**Figure 5:**
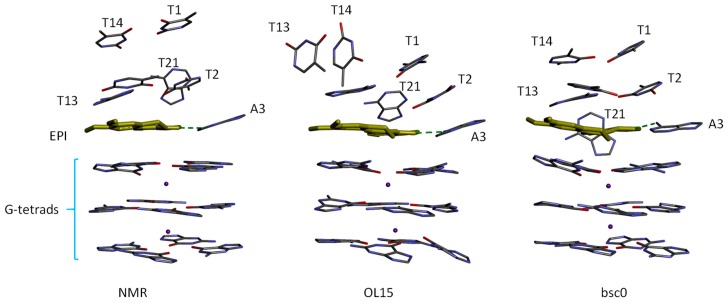
Comparison of the (**left**) NMR structure with the MD snapshots taken at 10 ns from the simulations using (**center**) OL15 and (**right**) bsc0 force field parameters. For clarity, only the nucleotide bases forming the 5′ binding site and the G-tetrads are displayed. The green dashed line indicates the EPI-A3 hydrogen bond.

Although there is a good correlation between the ranking order of ΔG(MM-GBSA) obtained using OL15 and the experimental binding affinities, the snapshots from the MD simulations reveal that the simulated-structure of the ligand binding site region is distorted compared with the NMR structure of the EPI-wtTel26 G4 complex [6], particularly in the simulations using the OL15 parameters. Figure 5 shows the binding site structures according to NMR and simulations. It can be seen that the four-layered binding pocket found in the NMR structure is distorted in the OL15 simulation. In particular, in the NMR structure, the T13/T14 base planes are parallel to the plane of EPI aromatic ring system, whereas in the OL15-simulated structure, the T13/T14 bases are perpendicular to the EPI plane. Structural deviations from the experimental structure are also observed in the bsc0-simulated structure: For example, the orientation of the A21 base shifts by 45 degrees from that in the NMR structure (Figure 5). However, compared with OL15-simulated structure, the binding pocket in the bsc0-simulated structure appears to be better preserved. Although we have observed structural deviations from the NMR structure of the EPI-wtTel26 G4 complex using both DNA force fields, we caution that these observations regarding the performance of the force fields may not necessarily apply to other systems, since MD simulations of DNA-ligand complexes can be sensitive to a number of factors, including the DNA topology in the ligand binding site as well as the nature of ligand. For example, Wu et al. [26] have used OL15 to accurately model the binding of BRACO19, a much larger ligand with the parallel stranded human telomeric G-quadruplex, which has a very different topology from the hybrid-2 human telomeric G4 studied here.

### 2.4. Thermodynamic Driving Force for the Protoberberine-wtTel26 Binding

**Figure 6:**
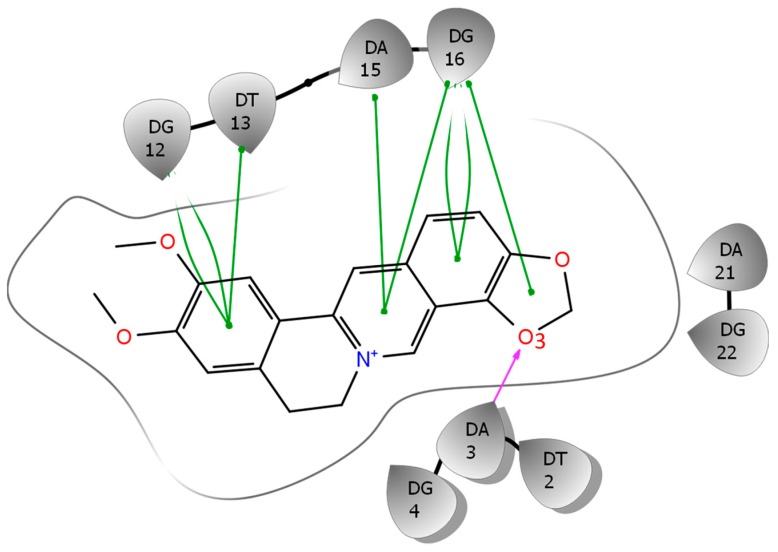
The ligand interaction diagram based on the NMR structure of the EPI-wtTel26 complex. The green lines represent stacking, and purple arrow indicates H-bonding interaction.

To gain further insights into the general thermodynamic driving force in the protoberberine-wtTel26 G4 binding, we examined the binding free energy contributions given by the MM-PB(GB)SA (Table 1). Since the structures simulated using bsc0 is in better agreement with the experimental structure, the energetics and trajectory analysis discussed below are based on the bsc0 results. It can be seen that the binding is favored by the ligand-DNA van der Waals interaction ΔE(vdw), the electrostatics interaction ΔE(elec), and to a lesser extent the buried surface area term ΔG(solv_np) (Table 1). The favorable ΔE(vdw) reflects the extensive stacking interaction between the ligands and the DNA binding pocket induced by the ligand: See Figure 6. The very large electrostatic interaction ΔE(elec) of ~ −550 kcal/mol arises mainly from the Coulomb attraction between the oppositely charged EPI (+1e) and DNA (−23e, including two channel K^+^ cations). Table 1 also shows that the binding is strongly opposed by the electrostatic desolvation free energy ΔG(solv_elec) which more than cancels out the large electrostatic interaction ΔE(elec). As a result, the net electrostatic contribution ΔG(total_elec) to binding is unfavorable, at around +20 kcal/mol. Overall, the binding is driven by the net nonpolar interaction, ΔG(total_np), which is the sum of ΔE(vdw) and ΔG(solv_np) and opposed by the net electrostatic contribution ΔG(total_elec). 

### 2.5. Binding Free Energy Components and MD Trajectory Analysis Help Rationalize the Ligand Selectivity

**Figure 7:**
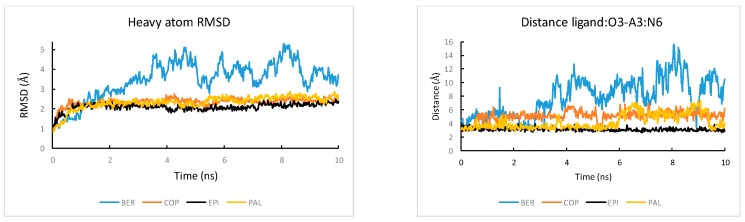
The MD simulated time courses of (**left**) the RMS deviation from the starting structures of the complex and (**right**) the ligand:O3-A3:N6 distance in the complexes of the four ligands bound to the wtTel26 G4.

Among the four alkaloids, EPI has the most favorable ligand-DNA van der Waals interaction ΔE(vdw) (−68.1 kcal/mol) and electrostatics interaction ΔE(elec) (−579.2 kcal/mol). Therefore, the ligand selectivity that favors EPI over other compounds is determined by both the more optimal van der Waals packing and ligand-DNA electrostatics interactions between EPI and wtTel26. To understand the structural origin of these two factors, we look at the ligand-DNA interaction diagram abstracted from the NMR structure for the EPI-wtTel26 complex: See Figure 6. The EPI-wtTel26 complex is stabilized by a number of stacking interactions from G12/G13 and A15/G16; these are reflected by the favorable ΔE(vdw) of −68.1 kcal/mol for EPI (Table 1). The PAL and COP have weaker ΔE(vdw) of −65.2 kcal/mol and −59.4 kcal/mol, respectively, while BER has the weakest ΔE(vdw) at −46.8 kcal/mol. Examining the MD trajectories, we find that these ΔE(vdw) values correlate with the structural stability of the ligand-DNA complex (Figure 7). As shown in Figure 7, the root-mean-squared deviations (RMSD) from the starting complexes are in the order EPI < PAL ≤ COP ≪ BER, i.e., consistent with the decreasing van der Waals packing seen in the ΔE(vdw) values. The larger RMSD fluctuations in the MD trajectory of BER (Figure 7, left panel) are also consistent with its weaker experimental binding affinity compared with the other compounds (Figure 3).

Next, we look at the structural factors that differentiate the electrostatics interaction ΔE(elec) among the different ligands (Table 1). The order of ΔE(elec) is EPI ≤ PAL < COP ≪ BER. The ΔE(elec) for BER is much weaker than other ligands by more than 20 kcal/mol, which is clearly related to the instability in the binding pocket containing BER: See Figure 7, left panel. Among the other three ligands, the EPI exhibits the strongest ΔE(elec), which can be attributed to the H-bond between the EPI:O3 and N6 of A3 (Figure 6) that appears to play an important role in the more favorable electrostatic interaction ΔE(elec) in EPI relative to that in COP and PAL. To investigate the stability of the EPI:O3-A3:N6 H-bond in the MD simulations, we examine the distance between the EPI:O3-A3 along the MD trajectories: See Figure 7, right panel. Among the four compounds, only EPI maintains EPI:O3-A3:N6 H-bond throughout the entire MD trajectory (formed in ~92% of the time). In the PAL-wtTel26 complex, the PAL:O3-A3:N6 hydrogen bond is present in less than 35% of the time. This is consistent with the fact that the electrostatic interaction ΔE(elec) for the PAL binding, despite its relatively large fluctuation, is on average about 5 kcal/mol weaker compared with that in the EPI binding (Table 1). In the complexes containing COP and BER, the ligand-A3 H-bond is completely absent, which contributes to their significantly weaker ΔE(elec) relative to that of EPI and PAL (Table 1).

### 2.6. NMR Titration Shows that Among the Protoberberines Studied Here Only EPI Binds Specifically to the Hybrid-2 Human Telomeric G4

**Figure 8:**
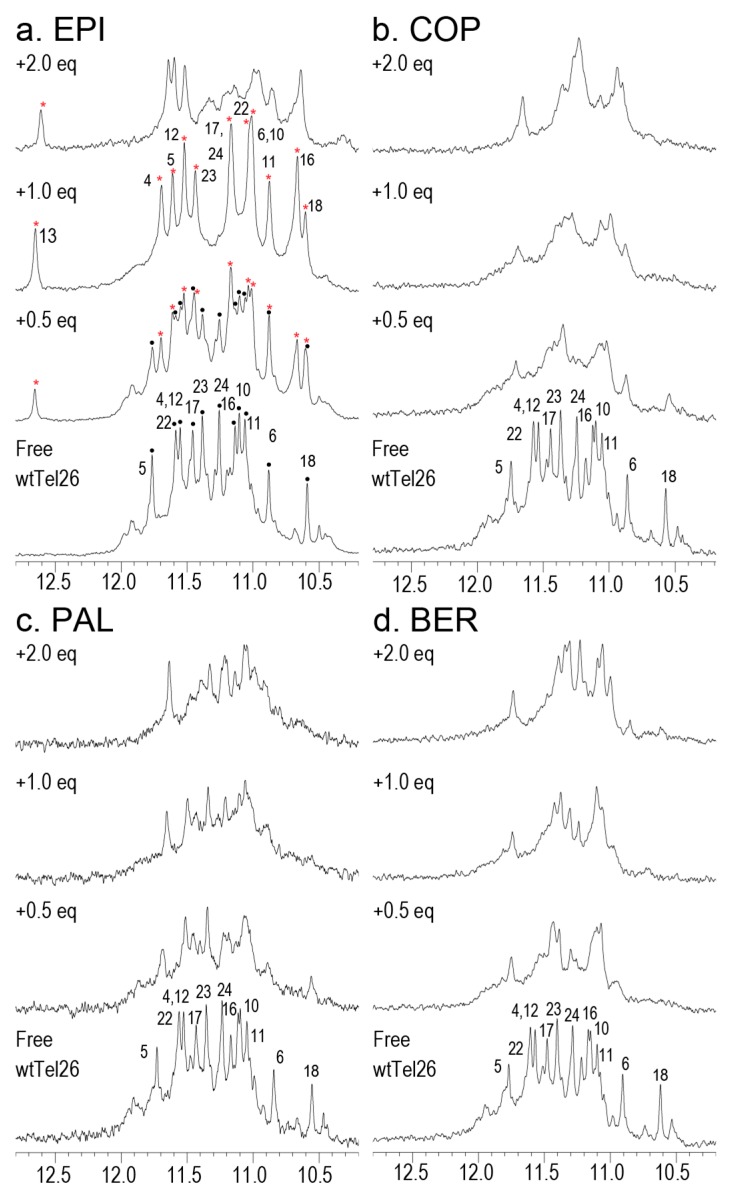
The ^1^H-NMR imino region in the presence of protoberberine alkaloids titration to wtTel26: (**a**) EPI, (**b**) COP, (**c**) PAL, and (**d**) BER. The assignments of the free wtTel26 in K^+^-containing solution are shown. Also shown in (**a**) are the assignments of EPI-wtTel26 complex (red asterisks) and free DNA (black dots).

We conducted 1D ^1^H-NMR titration experiments for the four protoberberine alkaloids to wtTel26. Figure 8 shows the ^1^H-NMR imino region of titrating the four compounds BER, COP, EPI and PAL to wtTel26. It can be seen that only EPI forms well-defined complex with wtTel26 as demonstrated by its well-resolved spectra lines upon addition of ligand to free DNA. The observation of peaks corresponding to both the free DNA and the EPI:wtTel26 at 0.5eq EPI complex indicates EPI binding has a slow exchange rate on the NMR time scale, which is characteristic of ligands binding with high affinity and specificity. In contrast, the addition of the other compounds BER, COP or PAL results in the broadening of peaks corresponding to free DNA, indicative of less-specific binding which occurs with a medium exchange rate on the NMR time scale. Notably, the peak occurring at 12.6 ppm in the wtTel26:EPI complex, corresponding to the formation of the T13 hydrogen-bonded capping structure in the EPI complex, was not observed in the other complexes [6]. Taken together, the NMR results suggest that compared with EPI, the other compounds exhibit less-optimal binding interactions with wtTel26, consistent with the calculated binding free energies (Table 1 and Table 2). 

### 2.7. The More Rigorous DDM Calculations Make Incorrect Predictions for the Top Binder of the Hybrid-2 Human Telomeric G4

We have also evaluated the performance of the more rigorous DDM method in estimating the absolute binding free energy $\Delta G_{bind}^{o}$ for protoberberine-wtTel26 G4 binding (Table 3). The DDM calculation is carried out using the bsc0 force field. The performance of the DDM results is mixed: On the one hand, the absolute magnitudes of the calculated $\Delta G_{bind}^{o}$ are quite reasonable. For example, the calculated $\Delta G_{bind}^{o}$ for EPI:wtTel26 binding is −8.9 ± 0.4 kcal/mol, which is similar in magnitude to experimental free energy of −10.7 ± 0.02 kcal/mol. On the other hand, the DDM calculation incorrectly predicts EPI as the second-best binder after COP. Thus, the calculated $\Delta G_{bind}^{o}$ exhibits a poor correlation with the experimental binding free energies with R^2^ = 0.17. One potential challenge in estimating the binding free energy of protoberberines with the human telomeric G4 could come from the large conformational change induced by the ligand binding (Figure 2), which could be one source of error in the DDM calculation. This is because the an accurate DDM calculation requires correctly sampling the entire alchemical pathway connecting the complexed and dissociated states, which includes many intermediate states. In the wtTel26 G4, the binding site region near the 5′-end adopts completely different conformations in the complexed [6] and the unbound state [27] (Figure 2); and sampling reversibly such large reorganization in nanoseconds MD simulation can be challenging. In contrast, the MM-PB(GB)SA method requires only the two end-states, the complexed and unbound states, to be sampled accurately; there is no need to sample reversibly the many intermediate states along the alchemical pathway connecting the two end points. This could explain why the results from the more empirical MM-PB(GB)SA method yield better agreement with the experiments for these DNA-ligand complexes. 

## 3. Materials and Methods

### 3.1. Fluorescence Measurements of the Binding Dissociation Constant

Fluorescence spectra were acquired using a Jasco-FP8300 spectrofluorometer (Jasco Inc., Easton, MD, USA) equipped with a temperature-controlled circulator. Fluorescence was measured in a quartz cell with path length of 1 cm, using an excitation wavelength of 377 nm and an emission spectra scan from 520–600 nm. The titration experiments were carried out at protoberberine concentrations of 0.2 μM in 100 mM K^+^. wtTel26 DNA at the specified concentrations was titrated into the four protoberberine solutions, respectively, and the resulting solutions were incubated for 2 min before fluorescence measurements were taken. The disassociation constant K_d_ was calculated using GraphPad Prism software (San Diego, CA, USA fitting of an equation: A = A_min_ + (A_max_ − A_min_) [(P_T_ + O_T_ + K_d_) − [((P_T_ + O_T_ + K_d_)^2^ − (4P_T_O_T_))^1/2^]/(2O_T_), where A represents the fluorescence intensity of the protoberberines bound to wtTel26 DNA. Both A_max_ and A_min_ are fit in addition to K_d_. The protoberberine concentration, O_T_, was held constant, and P_T_, the total complex concentration is the independent variable, varying with each measurement of A.

### 3.2. NMR Titration

wtTel26 DNA oligonucleotides were synthesized and purified as previously described [6] using commercially available reagents. Oligonucleotide samples were prepared to a final concentration of 100–250 µM in buffer solution containing 25 mM potassium phosphate, 70 mM potassium chloride and 90/10% H_2_O/D_2_O at pH 7. Samples were heated to 95 °C for 5 min then cooled slowly to room temperature for G-quadruplex formation. wtTel26 DNA was quantified by UV/Vis spectroscopy at 260 nm using its calculated extinction coefficient (261,200 L mol^−1^ cm^−1^). Ligand stock solutions were prepared at 40 mM in deuterated DMSO and titrated into the DNA solution to the desired concentrations for the complex samples. NMR experiments were conducted using a Bruker DRX-600 spectrometer or AV-500 (Billerica, MA, USA) spectrometer with cryoprobe. All experiments were performed using Watergate water suppression. 1D ^1^H-NMR spectra were collected at 25 °C. 

### 3.3. MD Setup and MM-PB(GB)SA Calculation

The starting structure for the molecular dynamics simulations is the solution NMR structure of the EPI-hybrid-2 human telomeric G-quadruplex (PDB entry 6ccw). The initial structures of the ligand-DNA complexes containing other three ligands (BER, COP and PAL) are modeled based on the NMR structure of the EPI-G4: note that these three compounds share the same heterocyclic core of the EPI; and they differ only in a methylenedioxy moiety and two methoxy groups located at the two ends (Figure 1). Therefore, starting from the NMR structure of the EPI-wtTel26 complex (Figure 2), the bound structures of the three weaker ligands are obtained by manually modifying the bonds at the two ends of the EPI structure in the binding pocket. Although there are no direct experimental information about the binding modes of these three weaker ligands BER, COP and PAL, the calculated binding free energies using the simulations starting from the modeled initial structures of the complexes correlate well with the experimental binding affinity (Figure 4). This indirectly supports the modeled initial structures of the three weaker compounds. 

The MM-PB(GB)SA binding free energy simulations were performed using the AMBER16 program [28]. As discussed earlier, the AMBER parm99bsc0 [23] force field and the more recent OL15 [24] modifications are used to model the G-quadruplex DNA in aqueous solutions. The four protoberberine compounds are modeled by the Amber GAFF parameters set [29] and the AM1-BCC charge model [30]. A truncated octahedral box containing TIP3P [31] water molecules was used to solvate the ligand-DNA complexes. The solvent box is set up to ensure that the distance between solute atoms from nearest walls of the box is at least 10 Å. K^+^ ions are added to the solvent box to maintain charge neutrality. The electrostatic interactions were computed using the particle-mesh Ewald (PME) method [32] with a real space cutoff of 10 Å and a grid spacing of 1.0 Å. A 2 fs time step is used for MD simulations. Before the production run, the system is equilibrated in a number of steps. First the energy minimized system is heated from 0 K to 300 K in 200 ps, while the solute atoms are restrained with a force constant k = 10 kcal/Å^2^ mol. Then the system is equilibrated for 8 ns with decreasing harmonic restraints: 2 ns with k = 5 kcal/Å^2^ mol, 2 ns with k = 1 kcal/Å^2^ mol, 2 ns with k = 0.25 kcal/Å^2^ mol, and 2 ns with k = 0.05 kcal/Å^2^ mol. Finally, a production MD of 10 ns is run in the NPT ensemble using the Langevin thermostat and isotropic position scaling for constant pressure (NTP = 1).

The MM-PB(GB)SA binding free energy [10,12] is computed using the one-trajectory protocol implemented by the MMPBSA.py [33] in the Amber16 release (The one-trajectory MM-PBSA is generally considered more robust compared with the three-trajectory MM-PBSA approach). Briefly, the binding free energy ΔG_bind_ is computed as the difference between the free energy of the complex and that of the two receptor and ligand

Δ*G_bind_* = *G*(*complex*) − *G*(*receptor*) − *G*(*ligand*) (1)

The free energy of species *X*, *G*(*X*), is approximated as

*G(X)* = *E(bonded)* + *E(elec)* + *E(vdw)* + *G(solv_elec)* + *G(solv_np)* − *TS(solute)* (2)

Combining Equations (1) and (2) and using the one-trajectory approximation yields

Δ*G_bind_* ≈ Δ*E(vdw)* + Δ*E(elec)* + Δ*G(solv_elec)* + Δ*G(solv_np)* − *T*Δ*S* (3)

The solute entropy term *T*Δ*S* is sometimes approximated by normal mode entropy [25], but such treatment rarely leads to improvement in the correlation with experiments. In this work we do not include the solute entropy term *T*Δ*S* in estimating Δ*G_bind_*. The electrostatic solvation free energy contribution Δ*G(solv_elec)* in Equation (3) is approximated using the continuum electrostatics Poisson-Boltzmann (PB) or Generalized Born (GB) approaches. The nonpolar solvation free energy Δ*G(solv_np)* is approximated by a solvent accessible surface area term. 

The OBC-GB [34] (igb = 2 in AMBER) model is used to compute the MM-GBSA energy, with the ionic concentration set to 0.1 M. The mbondi2 radii set was used for the atomic radii in GB.

### 3.4. DDM Calculation

In DDM [13,14,15,35], the absolute binding free energy is computed using

(4) $$\Delta G_{bind}^{o}=-\Delta G\left(complex\right)+\Delta G\left(solvent\right)+\Delta G_{restr–on}$$

Here ΔG(complex) includes the free energy of turning on a set of geometrical restraint when the ligand is bound [14], as well as the free energy of turning off the ligand interactions with its environment. ΔG(solvent) is the free energy of turning off the ligand-solvent interactions when the ligand is in the bulk solution. In addition, $\Delta G_{restr–on}$ is the free energy of turning on the set of geometrical restraints for an alchemically decoupled ligand. While ΔG(complex) and ΔG(solvent) are computed using simulation, the $\Delta G_{restr–on}$ is computed analytically [35]. 

The DDM calculation is performed using the GROMACS program [36,37]. The DNA molecule is described using the bsc0 force field and the ligands are described using the Amber GAFF parameters set [29] and the AM1-BCC charge model [30]. The detailed DDM protocol used in this work has been described previously [14]. 

## 4. Conclusions

We performed binding free energy calculations, fluorescence binding affinity measurements, and NMR titrations to investigate the underlying molecular basis for the ligand selectivity in the recognition of the hybrid-2 human telomeric G-quadruplex by protoberberines. The ligand molecules studied here have similar chemical structures but displayed markedly different binding affinities for the hybrid-2 human telomeric G4 (Figure 3). The binding free energies computed using the MM-PB(GB)SA are consistent with experimental binding affinity measurements and NMR titration experiments performed in this study. The results show that, in order to reproduce the experimental ligand selectivity in computation, it is crucial to account for the conformational flexibility in the ligand-DNA complexes in the binding free energy calculation. The analysis of the MD trajectories shows that, compared with other compounds, EPI has more optimal interactions with the 5’-pocket, including the high occupancy of the intermolecular H-bond with A3 in the binding site. We have examined the possible force field dependence of the binding free energy simulation and found structural deviations from the NMR structure in the binding site region in the simulations performed with both the more recent OL15 and the older bsc0 DNA force fields. While the performance of the OL15 force field has been examined on lateral and diagonal loops of the apo human telomeric G4 in extended simulations [21], the force field has yet to be extensively examined for simulating various loop conformations where ligands can bind, such as the one shown in this study. We have also found that, in the presence of very large ligand-induced conformational reorganization, the alchemical pathway method DDM may suffer from sampling problems, whereas the more empirical end-point method MM-PB(GB)SA actually performed better in capturing the ligand selectivity in these DNA-ligand systems. This study provides insights into the molecular determinants underlying the recognition of protoberberines by the human telomeric G4, and shed lights on the strengths and limitations of various binding fee energy methods and the DNA force fields currently used in the structure-based drug discovery targeting G-quadruplex DNA.

## Figures and Tables

**Table 1:** MM-GBSA binding free energies and their components computed using the bsc0 force field [23]. Units: kcal/mol.

Ligand	ΔE(vdw)	ΔE(elec)	ΔG(solv_elec)	ΔG(solv_np)	ΔG(MM-GBSA) ^a^	ΔG(total_elec) ^a^	ΔG(total_np) ^a^
BER	−46.8 ± 0.1	−539.1 ± 10.3	554.9 ± 11.5	−3.0 ± 0.1	−33.9 ± 1.2	15.9 ± 1.1	−49.8 ± 0.0
COP	−59.4 ± 0.4	−561.1 ± 0.2	582.0 ± 0.8	−3.6 ± 0.2	−42.1 ± 0.5	20.9 ± 1.0	−63.0 ± 0.5
EPI	−68.1 ± 0.2	−579.2 ± 0.5	598.0 ± 0.6	−4.0 ± 0.0	−53.3 ± 0.4	18.8 ± 0.1	−72.1 ± 0.2
PAL	−65.2 ± 2.4	−573.8 ± 5.0	595.6 ± 5.2	−4.5 ± 0.1	−47.9 ± 2.4	21.8 ± 0.2	−69.7 ± 2.5

^a^ The binding free energy ΔG(MM-GBSA) = ΔE(vdw) + ΔE(elec) + ΔG(solv_elec) + ΔG(solv_np) = ΔG(total_elec) + ΔG(total_np). ΔE(vdw): the van der Waals energy contribution; ΔE(elec): the direct receptor-ligand electrostatic interaction energy; ΔG(solv_elec): the electrostatic component of the solvation free energy; ΔG(solv_np): the nonpolar component of the solvation free energy; ΔG(total_elec): the total electrostatic contribution to binding free energy, which comprises ΔE(elec) and ΔG(solv_elec), i.e., ΔG(total_elec) = ΔE(elec) + ΔG(solv_elec); ΔG(total_np): the total nonpolar contribution to binding free energy, which consists of ΔE(vdw) and ΔG(solv_np), i.e., ΔG(total_np) = ΔE(vdw) + ΔG(solv_np).

**Table 2:** MM-GBSA Binding free energies and their components computed using the Amber force field OL15 parameters. Units: kcal/mol.

Ligand	ΔE(vdw)	ΔE(elec)	ΔG(solv_elec)	ΔG(solv_np)	ΔG(MM-GBSA)	ΔG(total_elec)	ΔG(total_np)
BER	−60.7 ± 2.6	−557.2 ± 5.9	577.4 ± 6.2	−4.1 ± 0.1	−44.6 ± 2.2	20.2 ± 0.3	−64.8 ± 2.5
COP	−60.9 ± 1.7	−566.5 ± 2.9	585.4 ± 2.6	−3.4 ± 0.0	−45.3 ± 2.1	18.9 ± 0.3	−64.3 ± 1.7
EPI	−64.7 ± 0.9	−573.4 ± 3.2	592.4 ± 3.9	−4.1 ± 0.0	−49.9 ± 0.2	19.0 ± 0.7	−68.8 ± 0.9
PAL	−67.2 ± 0.4	−581.3 ± 1.3	605.4 ± 5.2	−4.4 ± 0.1	−47.5 ± 0.8	24.1 ± 0.4	−71.6 ± 0.5

**Table 3:** The DDM-calculated absolute binding free energies $\Delta G_{bind}^{o}$ of ligand binding to wtTel26. Unit: kcal/mol.

Ligand	−ΔG(complex)	ΔG(solvent)	$\Delta G_{restr–on}$	$\Delta G_{bind}^{o}$ ^a^	$\Delta G_{bind}^{o}\left(expt\right)$ ^a^
BER	−42.8 ± 0.5	27.9 ± 0.0	7.3	−7.6 ± 0.5	−7.82 ± 0.06
COP	−45.9 ± 0.8	28.5 ± 0.1	7.3	−10.1 ± 0.8	−8.89 ± 0.1
EPI	−43.3 ± 0.4	27.1 ± 0.0	7.3	−8.9 ± 0.4	−10.70 ± 0.02
PAL	−40.3 ± 0.3	24.6 ± 0.1	7.3	−8.4 ± 0.3	−8.41 ± 0.01

^a^$\Delta G_{bind}^{o}=-\Delta G\left(complex\right)+\Delta G\left(solvent\right)$ + $\Delta G_{restr–on}$; ^b^ Obtained from the experimentally determined K_d_: see Figure 3.
